# Supplementary material for: APC/CFZR-1 Controls SAS-5 Levels To Regulate Centrosome Duplication in Caenorhabditis elegans
Source: G3 (Bethesda). 2017 Oct 13;7(12):3937–46. doi: 10.1534/g3.117.300260 (PMC5714490; doi:10.1534/g3.117.300260)
Supplement: Supplementary file 1 [file 3937FileS1.pdf]

Supplemental Materials

APC/C^{FZR-1} Controls SAS-5 Levels to Regulate Centrosome Duplication in *Caenorhabditis elegans*

Jeffrey C. Medley*, Lauren E. DeMeyer*, Megan M. Kabara* and Mi Hye Song*[,1]

* Department of Biological Sciences, Oakland University, Rochester, MI 48309, USA.
[1] To whom correspondence should be addressed.
Contact Information: msong2@oakland.edu

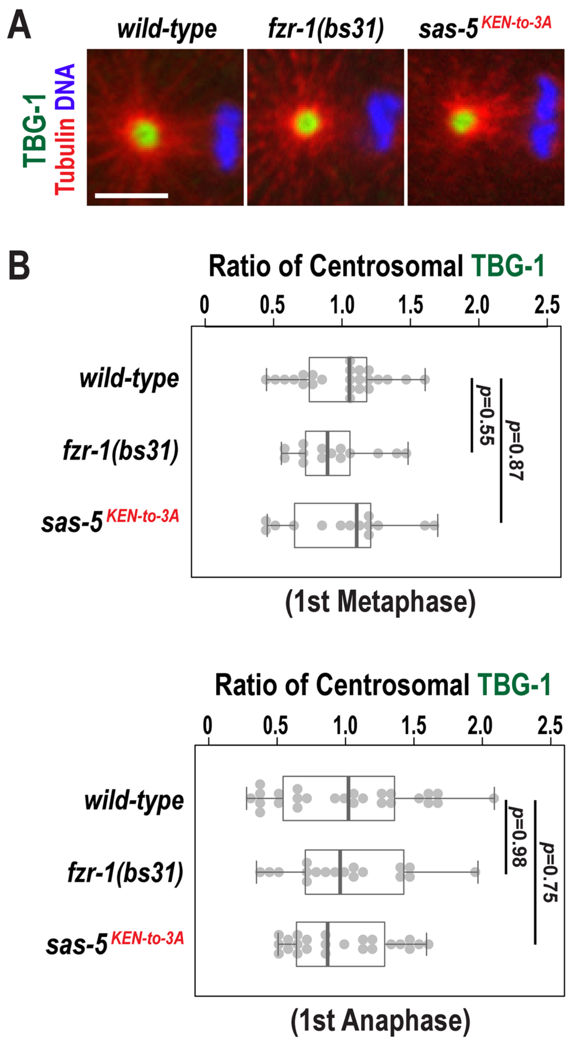

Figure S1. Centrosome-associated TBG-1 levels are unaffected in *fzr-1(bs31)* and *sas-5$^{KEN\text{-}to\text{-}3A}$* mutant embryos. (A) Centrosomes stained for TBG-1 (green) at the first metaphase. Bar, 5μm. (B) Quantification of TBG-1 levels at centrosomes during the first mitosis. TBG-1 levels are normalized to the average fluorescence intensity in wild-type (N2) control embryos. At the first metaphase, *fzr-1(bs31)* (0.94±0.28 fold, n=14; *p*=0.55) and *sas-5$^{KEN\text{-}to\text{-}3A}$* mutants (1.02±0.40 fold, n=14; *p*=0.87) have comparable centrosomal TBG-1 levels to the wild-type control (1.00±0.30 fold, n=24). At the first anaphase, centrosome-associated TBG-1 levels in both *fzr-1(bs31)* (0.99±0.42 fold, n=18; *p*=0.98) and *sas-5$^{KEN\text{-}to\text{-}3A}$* (0.96±0.36 fold, n=24; *p*=0.75) mutant embryos are similar to those of the wild-type control (1.00±0.52 fold, n=26). n is the number of centrosomes. Each dot represents a centrosome. Boxes ranges from the first through third quartile of the data. Thick bar indicates the median. Solid grey line extends 1.5 times the inter-quartile range or to the minimum and maximum data point.

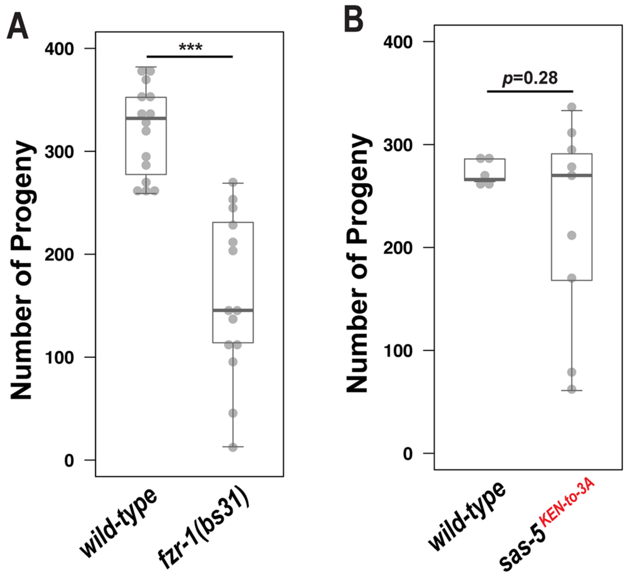

Figure S2. Brood size in *fzr-1(bs31)* and *sas-5*$^{KEN\text{-}to\text{-}3A}$ mutants. (A) *fzr-1(bs31)* mutants produce reduced brood size (158.4±78.4, n=14 hermaphrodites; $p<0.001$) compared to wild-type animals (319.6±43.9, n=15 hermaphrodites) grown at 24°. Note that *fzr-1(bs31)* mutants produce a wide range of distribution in brood size among 14 animals tested, which is also seen in *sas-5*$^{KEN\text{-}to\text{-}3A}$ mutants. (B) *sas-5*$^{KEN\text{-}to\text{-}3A}$ mutants display a slight reduction in brood size (222.7±99.7, n=9 hermaphrodites; $p=0.28$) compared to wild-type controls (273.4±11.5, n=5 hermaphrodites) grown at 24°. Compared to wild-type animals, *sas-5*$^{KEN\text{-}to\text{-}3A}$ mutant animals produce highly irregular number of progeny in the population of nine animals tested under the same condition. Each dot represents the total number of progeny produced by a single animal. Box ranges from the first through third quartile of the data. Thick bar indicates the median. Solid grey line extends 1.5 times the inter-quartile range or to the minimum and maximum data point.

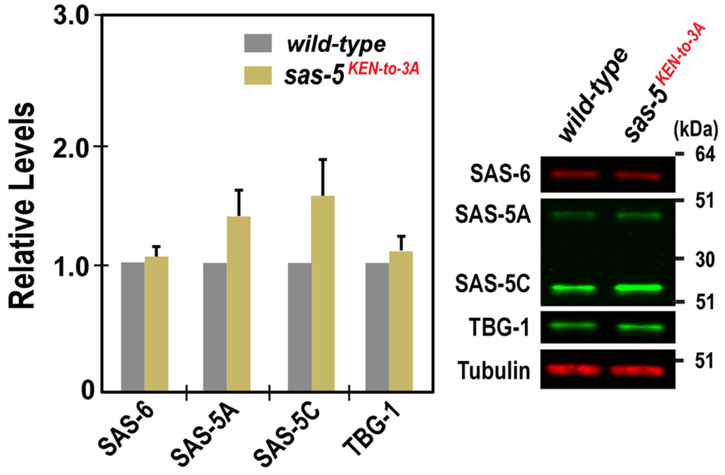

Figure S3. SAS-5 levels are increased in *sas-5*$^{KEN\text{-}to\text{-}3A}$ mutants. Quantitative western blot reveals that (left panel) ***sas-5***$^{KEN\text{-}to\text{-}3A}$ mutant embryos contain increased levels of both SAS-5 isoforms, SAS-5A (1.36±0.20 fold) and SAS-5C (1.52±0.28 fold), compared to wild-type (N2) control embryos. In contrast, there were no significant changes in either SAS-6 (1.05±0.04 fold) or TBG-1 (1.09±0.11 fold) levels between *sas-5*$^{KEN\text{-}to\text{-}3A}$ mutant and wild-type controls. Four biological samples and six technical replicates were used for the statistical analysis. Average values are presented and error bars are SD. (right panel) Representative western blot using embryonic lysates from *sas-5*$^{KEN\text{-}to\text{-}3A}$ mutants and wild-type (N2) animals. Tubulin was used as a loading control.

Table S1. List of *C. elegans* Strains Used in This Study

Name	Genotype	Origin
N2	*wild-type*	CGC
CB4856	*wild-type, Hawaiian variant*	CGC
CB120	*unc-4(e120) II*	Brenner 1974
CB128	*dpy-10(e128) II*	Brenner 1974
MJ57	*emb-1(hc57) III*	Schierenberg *et al.* 1980
MTU6	*fzr-1(bs31) II*	This study
MTU7	*mat-3(or180) III*	Golden *et al.* 2000
MTU8	*zyg-1(it25) II; mat-3(or180) III*	This study
MTU9	*zyg-1(it25) II; emb-1(hc57) III*	This study
MTU10	*unc-119(ed3) III; [unc-119(+); fzr-1p::fzr-1::gfp::fzr-1 3'UTR]*	This study<br>Sarov *et al.* 2012
MTU11	*sas-5(mhs357) [sas-5*$^{KEN\text{-}to\text{-}3A}$*] V*	This study
MTU12	*sas-5(mhs358) [sas-5*$^{KEN\text{-}to\text{-}3A}$*] V*	This study
MTU13	*zyg-1(it25) II; sas-5(mhs359) [sas-5*$^{KEN\text{-}to\text{-}3A}$*] V*	This study
MTU14	*zyg-1(it25) II; sas-5(mhs361) [SAS-5*$^{KEN\text{-}to\text{-}KEN}$*] V*	This study
MTU15	*zyg-1(it25) II; sas-5(mhs362) [sas-5*$^{KEN\text{-}to\text{-}3A}$*] V*	This study
OC13	*zyg-1(or409) II*	Kemp *et al.* 2007
OC14	*zyg-1(it25) II*	Kemphues *et al.* 1988
OC130	*zyg-1(it25) fzr-1(bs38) II*	Kemp *et al.* 2007
OC190	*unc-119(ed3) III; bsIs6 [unc-119(+), pie-1p::gfp::fzr-1]*	This study
OC201	*zyg-1(it25) fzr-1(bs31) II*	Kemp *et al.* 2007
OC481	*unc-119(ed3) III; bsIs15[pNP99; unc-119(+), tbb-1p::mCherry::tbb2-2::tbb-2 3'UTR*	Gift from O'Connell Lab<br>Medley *et al.* 2017
OC740	*bsSi15[pKO109; unc-119(+), spd-2p::spd-2::mCherry::spd-2 3'UTR] I*	Peel *et al.* 2017
SA250	*tjIs54[pie-1p::GFP::tbb-2 + pie-1p::2xmCherry::tbg-1 + unc-119(+)]; tjIs57[pie-1p::mCherry::his-48 + unc- 119(+)]*	Toya *et al.* 2010

Table S2. List of Oligonucleotides for CRISPR/Cas9 Genome Editing

Construct	Sequence (5'-3')
sas-5 (KEN-box) crRNA	UUCUGCUGUCUUGAUUGACG
dpy-10 crRNA<br>(Arribere *et al.* 2014)	GCUACCAUAGGCACCACGAG
SAS-5-KEN-AAA ssODN	CTAAACAGCAAGCGATCGAACCAGTTGAAAAAGACGCTG<br>CTGCTTTTCATGAGAGTCCTAGACAGTCTCGTCAACAAAA<br>GCCAGCTAGTAAAGTGAGAATTCAGATAAAAAATA
dpy-10 ssODN<br>(Arribere *et al.* 2014)	CACTTGAACTTCAATACGGCAAGATGAGAATGACTGGAAA<br>CCGTACCGCATGCGGTGCCTATGGTAGCGGAGCTTCACA<br>TGGCTTCAGACCAACAGCCTAT

Supplemental References

Arribere, J. A., R. T. Bell, B. X. Fu, K. L. Artiles, P. S. Hartman *et al.*, 2014 Efficient marker-free recovery of custom genetic modifications with CRISPR/Cas9 in *Caenorhabditis elegans*. Genetics 198**:** 837-846.

Brenner, S., 1974 The genetics of *Caenorhabditis elegans*. Genetics 77**:** 71-94.

Golden, A., P. L. Sadler, M. R. Wallenfang, J. M. Schumacher, D. R. Hamill *et al.*, 2000 Metaphase to anaphase (*mat*) transition-defective mutants in *Caenorhabditis elegans*. J Cell Biol 151**:** 1469-1482.

Kemp, C. A., M. H. Song, M. K. Addepalli, G. Hunter and K. O'Connell, 2007 Suppressors of *zyg-1* define regulators of centrosome duplication and nuclear association in *Caenorhabditis elegans*. Genetics 176**:** 95-113.

Kemphues, K. J., M. Kusch and N. Wolf, 1988 Maternal-effect lethal mutations on linkage group II of *Caenorhabditis elegans*. Genetics 120**:** 977-986.

Medley, J. C., M. M. Kabara, M. D. Stubenvoll, L. E. DeMeyer and M. H. Song, 2017 Casein kinase II is required for proper cell division and acts as a negative regulator of centrosome duplication in *Caenorhabditis elegans* embryos. Biol Open 6**:** 17-28.

Peel, N., J. Iyer, A. Naik, M. P. Dougherty, M. Decker *et al.*, 2017 Protein Phosphatase 1 Down Regulates ZYG-1 Levels to Limit Centriole Duplication. PLoS Genet 13**:** e1006543.

Sarov, M., J. I. Murray, K. Schanze, A. Pozniakovski, W. Niu *et al.*, 2012 A genome-scale resource for *in vivo* tag-based protein function exploration in *C. elegans*. Cell 150**:** 855-866.

Schierenberg, E., J. Miwa and G. von Ehrenstein, 1980 Cell lineages and developmental defects of temperature-sensitive embryonic arrest mutants in *Caenorhabditis elegans*. Dev Biol 76**:** 141-159.

Toya, M., Y. Iida and A. Sugimoto, 2010 Imaging of mitotic spindle dynamics in *Caenorhabditis elegans* embryos. Methods Cell Biol 97**:** 359-372.
